# Increasing Adoption Trends of Gastric Per-Oral Endoscopic Myotomy Over Gastric Electrical Stimulation for Refractory Gastroparesis: A Nationwide Analysis

**DOI:** 10.1016/j.gastha.2026.101101

**Published:** 2026-08-21

**Authors:** Mohamed H. Eldesouki, Youssef Hafez, Ahmed Ibrahim, Tareq Alsaleh, Omar Al Taani, Amol Sharma

**Affiliations:** 1 Department of Internal Medicine, New York Medical College at Saint Michael's Medical Center, Newark, New Jersey; 2 Division of Gastroenterology and Hepatology, Saint Louis University School of Medicine, Saint Louis, Missouri; 3 Division of Gastroenterology and Hepatology, Medical University of South Carolina, Charleston, South Carolina; 4 Department of Internal Medicine, AdventHealth Orlando, Orlando, Florida; 5 Department of Medicine, Allegheny Health Network, Pittsburgh, Pennsylvania

Refractory gastroparesis causes persistent debilitating symptoms, impaired quality of life, and frequent hospitalizations, with limited durable treatment options. About 5 million US adults report gastroparesis-like symptoms, and as many as 40% develop a medically refractory disease, with associated nutritional compromise and substantial healthcare utilization underscoring a significant unmet need for effective therapies.^1^, ^2^, ^3^, ^4^

Gastric electrical stimulation (GES) is a long-standing, neuromodulation-based intervention for refractory gastroparesis, receiving US Food and Drug Administration Humanitarian Use Device approval in 2000 for refractory nausea and vomiting, with mixed results of symptomatic benefit in randomized trials and no improvement of gastric emptying.^1^^,^^5^ More recently, gastric per-oral endoscopic myotomy (G-POEM) has emerged as an alternative pylorus-directed therapy following the seminal in-human description by Khashab et al in 2013, with growing guideline endorsement for carefully selected patients with documented delayed gastric emptying and predominant nausea and vomiting symptoms.^6^^,^^7^ However, comprehensive national-level data describing real-world adoption and penetration of these interventions remain limited.

The 2025 American Gastroenterological Association guideline conditionally recommends selective use of G-POEM, acknowledging that observational studies report more favorable results than sham-controlled data.^5^ The American College of Gastroenterology guideline assigns low-quality evidence and conditional strength to nearly all gastroparesis interventions, including GES and pyloric therapies.^8^ Observational and propensity-matched studies suggest therapeutic benefit with G-POEM, supporting its growing adoption at specialized centers.^9^^,^^10^ We therefore aimed to characterize national utilization patterns and temporal trends of GES and G-POEM among adults with gastroparesis using a large, US-based electronic health record database.

We performed a retrospective cohort analysis using the TriNetX US Collaborative Network, a federated electronic health record database comprising 67 US healthcare organizations (HCOs). Adult patients (≥18 years) with a diagnosis of gastroparesis were identified using International Classification of Diseases, 10th revision (ICD-10) code K31.84. Our primary comparison was between GES and G-POEM, the 2 dedicated procedural therapies aimed at modifying the gastroparesis pathophysiology; we also characterized the contemporaneous utilization of supportive and anatomy-altering interventions (venting gastrostomy, feeding jejunostomy, parenteral nutrition, and gastrectomy) for context, recognizing that these modalities serve nutritional and decompressive purposes rather than competing therapeutic indications. Patients undergoing GES or G-POEM were identified using relevant Current Procedural Terminology and ICD-10 Procedure Coding System codes (43,647, 43,881, 0DH60MZ, 0DH64MZ, 0DW64MZ, and 64,590 for GES; 43,999 and 0D878ZZ for G-POEM). The 2015 starting point was chosen because (a) it is the year of early US adoption of G-POEM following the seminal in-human description by Khashab et al^7^ (2013), and (b) the US transition to international classification of diseases, tenth revision, clinical modification/procedure coding system. on October 1, 2015, introduced procedure code 0D878ZZ—the only administrative code that specifically and reliably captures G-POEM. Incidence and prevalence of each intervention were assessed across consecutive 2-year intervals from 2015 through 2024. Temporal trends in procedural share were evaluated using log-linear regression on the proportion of dedicated procedural interventions accounted for by each modality, expressed as an annual percentage change (APC). Results were stratified by age, sex, race, ethnicity, US Census region, and HCO center type (academic vs nonacademic, as classified in TriNetX HCO metadata). All analyses were conducted within the TriNetX Analytics platform using deidentified data; institutional review board approval was not required.

Among 310,869 adults with gastroparesis identified between 2015 and 2024, 4599 patients (1.5%) underwent a dedicated procedural intervention, including 2708 (58.9%) who received GES and 1891 (41.1%) who underwent G-POEM. The proportion of patients undergoing GES declined from 78.2% in 2015–2016 to 54.6% in 2023–2024 (APC, −4.8%; *P* = .003), while G-POEM utilization increased from 21.8% to 45.4% over the same interval (APC, +10.2%; *P* = .005) (Figure). Demographic characteristics of patients undergoing each procedure are shown in Table. Other nonpharmacologic interventions queried for context showed parallel temporal increases across the study period; in 2023–2024, the incidence proportions in the source gastroparesis population were 0.81% for venting gastrostomy, 0.53% for feeding jejunostomy, 0.47% for parenteral nutrition, and 0.30% for gastrectomy, compared with 0.37% for GES and 0.31% for G-POEM. Geographic coverage spanned all 4 US Census regions, with a contrasting regional distribution between modalities: GES recipients were concentrated in the South (1361/2708; 50%) and Northeast (627; 23%), while G-POEM recipients were concentrated in the Northeast (803/1891; 43%, predominantly the Middle Atlantic division) and South (638; 34%). In stratified analyses by center type, both academic and nonacademic centers showed temporal increases in G-POEM share consistent with the overall cohort. Within the academic stratum (48 HCOs, 217,255 patients), G-POEM share rose from 21.3% in 2015–2016 to 41.4% in 2023–2024 (APC +9.2%/y, *P* = .012), with GES remaining the more frequently performed procedure throughout the study period. Within the nonacademic stratum (17 HCOs, 91,688 patients), G-POEM share rose from 24.4% to 57.1% over the same interval (APC +12.0%/y, *P* = .001), with G-POEM and GES procedural shares appearing to converge by 2021–2022.

**Figure:**
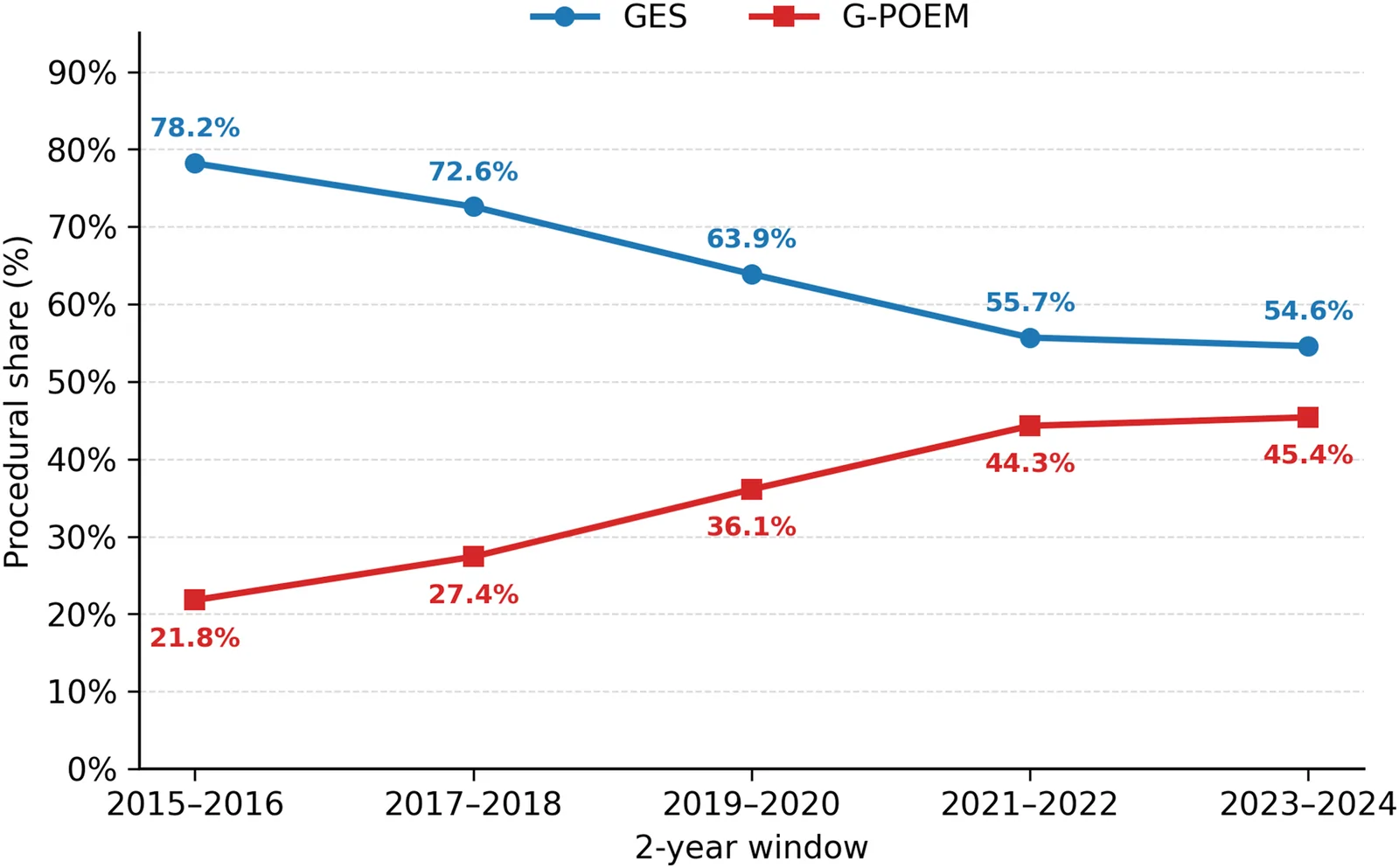
Temporal redistribution of gastric per-oral endoscopic myotomy and gastric electrical stimulation among adults undergoing dedicated procedural intervention for gastroparesis in the TriNetX US Collaborative Network, 2015–2024. The y-axis shows the percentage of dedicated procedural interventions accounted for by each modality in each 2-year window. GES share declined from 78.2% (2015–2016) to 54.6% (2023–2024) (annual percentage change [APC], −4.8%; *P* = .003), while G-POEM share rose from 21.8% to 45.4% over the same interval (APC, +10.2%; *P* = .005).

**Table:** Demographic Characteristics of Gastroparesis Patients Undergoing GES vs G-POEM (2015–2024)

Characteristic	GES (n = 2708)	G-POEM (n = 1891)
Age (y), mean ± SD	55 ± 15	57 ± 17
Sex		
Female	2249 (83.0%)	1386 (73.3%)
Male	459 (17.0%)	503 (26.6%)
Race		
White	2069 (76.4%)	1418 (75.0%)
Black or African American	420 (15.6%)	256 (13.5%)
Asian	20 (0.8%)	26 (1.4%)
American Indian/Alaska Native	14 (0.6%)	10 (0.5%)
Native Hawaiian/Pacific Islander	10 (0.4%)	10 (0.5%)
Other race	92 (3.4%)	49 (2.5%)
Unknown race	83 (3.1%)	123 (6.5%)
Ethnicity		
Hispanic or Latino	270 (10.0%)	135 (7.1%)
Not Hispanic or Latino	2083 (76.9%)	1473 (77.9%)
Unknown ethnicity	355 (13.1%)	283 (15.0%)

Values are n (%) within each procedure cohort unless otherwise specified. Demographic characteristics are reported descriptively for each procedure cohort; a formal between-group statistical comparison was not performed.

We demonstrate a marked shift in refractory gastroparesis management, with declining GES utilization and increasing G-POEM adoption that varies by region and center type. Accelerating G-POEM uptake likely reflects gastroenterologists' procedural predisposition, the success of POEM for achalasia, and growth of third-space endoscopy rather than definitive outcome superiority. Compared with surgical pyloroplasty, G-POEM offers shorter procedure times, minimal blood loss, and reduced length of stay, lowering operating room and inpatient resource use.^8^ G-POEM also aligns with the pylorus-directed paradigm reinforced by objective EndoFLIP measurements of pyloric compliance.^11^ The female and predominantly white composition of both procedural cohorts is consistent with the established epidemiology of gastroparesis.^12^, ^13^, ^14^

Multiple factors contribute to declining GES utilization, including limited evidence of consistent efficacy, device-related complications, a lack of clear response predictors, perceived investigational status, and declining specialist experience.^5^ GES is indicated for drug-refractory nausea and vomiting but not for persistent abdominal pain, limiting its applicability to mixed-symptom phenotypes.^1^^,^^5^ In contrast, pylorus-directed G-POEM may address broader symptom constellations including bloating, early satiety, and postprandial fullness, contributing to preferential referral of mixed-phenotype patients toward endoscopic approaches.^6^ Heterogeneous payer-coverage policies likely also contribute: GES coverage remains variable across commercial payers and frequently requires documented refractoriness to standard therapy, while G-POEM has historically been reimbursed under unlisted-procedure codes. Network meta-analysis data nonetheless indicate that GES may provide sustained benefit in carefully phenotyped patients, supporting its continued role rather than obsolescence.^9^

Limitations include the retrospective design and reliance on administrative coding, which may introduce misclassification bias. TriNetX does not provide granular clinical detail, precluding assessment of symptom severity, gastroparesis etiology, gastric emptying, response durability, or adverse events. The federated platform does not expose individual-center identifiers, precluding restriction of the analysis to centers offering both procedures or precise identification of provider-level equivalence; we approximated this by stratifying on center type. Standard incidence-and-prevalence outputs do not allow reliable enumeration of patients undergoing both procedures or their sequencing within the network's privacy-preserving architecture. Payer-level data (prior authorization, approval rates, denials, appeals) are not captured and could not be assessed; differential coverage between GES and G-POEM may influence access in ways we cannot directly measure. Unmeasured confounding related to referral patterns, center expertise, and patient selection may also have influenced observed trends.

In conclusion, we demonstrate a marked shift toward endoscopic-first management of refractory gastroparesis, with regional and center-type heterogeneity that reflects access expansion as well as practice change. These evolving practice patterns underscore the need for large, prospective, randomized, sham-controlled trials of G-POEM with standardized symptom phenotyping, durability endpoints, and patient-centered outcomes.
